# Genetic manipulation of bacteriophage T4 utilizing the CRISPR-Cas13b system

**DOI:** 10.3389/fgeed.2024.1495968

**Published:** 2024-12-19

**Authors:** Yuvaraj Bhoobalan-Chitty, Mathieu Stouf, Marianne De Paepe

**Affiliations:** ^1^ Université Paris-Saclay, INRAE, AgroParisTech, Micalis Institute, Jouy-en-Josas, France; ^2^ Department of Biology, University of Copenhagen, Copenhagen, Denmark

**Keywords:** T4 genome editing, CRISPR-Cas13, T4-YFP, single-cell, bacteriophage T4

## Abstract

CRISPR-Cas type II and type V systems are inefficient in modifying bacteriophage T4 genome, due to hypermodification of its DNA. Here, we present a genome editing technique for bacteriophage T4 using the type VI CRISPR-Cas system. Using BzCas13b targeting of T4 phage, we were able to individually delete both T4 glucosyl transferase genes, *α-gt* and *β-gt*. Furthermore, we employed this method to mutate two conserved residues within the T4 DNA polymerase and to introduce the yellow fluorescent protein (YFP) coding sequence into T4 phage genome, enabling us to visualize phage infections. This T4 genome editing protocol was optimized to generate recombinant phages within a 6-hour timescale. Finally, spacers homologous to a variety of T4 genes were used to study the generality of Cas13b targeting, revealing important variability in targeting efficiency. Overall, this method constitutes a rapid and effective means of generating specific T4 phage mutants, which could be extended to other T4-like phages.

## Introduction

Since their discovery, the ability of Clustered Regularly Interspaced Short Palindromic Repeats and associated genes (CRISPR-Cas) systems to sequence-specifically cleave nucleic acids has been exploited for the genetic manipulation of organisms across all domains of life (Ishino et al., 2018; Wang and Doudna, 2023). CRISPR-Cas are divided into Class 1 and Class 2 systems based on the complexity of the ribonucleoprotein interference complexes, and are further divided into types I, III, IV (belonging to Class 1) and types II, V, VI (belonging to Class 2) (Makarova et al., 2015). The type II CRISPR-Cas system Cas9, with its ability to introduce double-strand DNA breaks, is the most widely utilized CRISPR-Cas system for genome editing.

Bacteriophage T4, infecting *Escherichia coli*, is one of the best-studied bacteriophage. Historically, T4 mutants were generated based on induced (Latarjet, 1954; Latarjet, 1949) or spontaneous (Reha-Krantz and Bessman, 1981) mutagenesis followed by selective pressure, such as temperature sensitivity or resistance to phosphonoacetic acid (Edgar and Lielausis, 1964; Edgar et al., 1964; Epstein et al., 1963); all laborious, time-consuming and non-specific mutagenesis techniques. Specific gene modification techniques using homologous recombination with a template plasmid have also been largely used, but have remained laborious to purify the recombinant in the absence of an easily selectable phenotype, and did not permit to obtain deleterious mutations (Mattson et al., 1977; Ramirez-Chamorro et al., 2021). Over the past decade, phage genome editing techniques utilizing homologous recombination coupled with selection of recombinants by CRISPR-Cas targeting have been developed for *E*. *coli* bacteriophages T3, T4, T5, T7, lambda, P1 and *Klebsiella* phages (Ramirez-Chamorro et al., 2021; Kiro et al., 2014; Tao et al., 2017; Shen et al., 2018; Duong et al., 2020a; Dong et al., 2021; Isaev et al., 2022). However, these techniques rely on double-strand DNA targeting CRISPR-Cas systems, Cas9 and Cpf1. CRISPR-Cas type II and type V dependent genome editing of T-even phages has been shown to be irregular due to hypermodification of the cytosine residues in the phage DNA (Tao et al., 2017; Dong et al., 2021; Vlot et al., 2018; Duong et al., 2020b; Yaung et al., 2014; Bryson et al., 2015; Liu et al., 2020). Hypermodification of cytosines in T4 was previously demonstrated to be important in distinguishing between host and phage genetic material, enabling phage protection against restriction-modification systems (Revel and Luria, 1970; Hattman and Fukasawa, 1963), phage T4 induced host genome degradation, and in ensuring phage transcriptional specificity (Kutter et al., 1981).

Cas13, belongs to the type VI CRISPR-Cas system and it is unique in that while retaining the sequence specificity of Class 2 CRISPR-Cas systems, it recognizes RNA targets instead of DNA like the type II and type V CRISPR-Cas systems (Shmakov et al., 2015; Abudayyeh et al., 2016). Apart from cleaving target RNA, based on sequence specificity, it also contains a non-specific trans-RNA collateral cleavage activity, regulated by the protospacer containing target RNA (Abudayyeh et al., 2016; Meeske et al., 2019). To date, type VI CRISPR-Cas system has been further classified into four Cas13 phylogenetic subtypes, from Cas13a to Cas13d, presenting limited amino-acid sequence similarity. RNA targeting by CRISPR-Cas13 system is dependent on the crRNA efficiency (Guan et al., 2022), expression level of the target transcript (Vialetto et al., 2022) and protospacer-flanking site (PFS) requirements (Smargon et al., 2017; Meeske and Marraffini, 2018; O'Connell, 2019). Recently, the Cas13a system was repurposed for genome editing in phage T4 (Adler et al., 2022) and *Pseudomonas aeruginosa* nucleus forming Jumbo phage ΦKZ (Guan et al., 2022), whose DNA is protected from nucleases. As CRISPR-Cas13 subtypes present differences in binding and cleavage specificity, we attempted to exploit the diversity amongst CRISPR-Cas13 system to expand the toolbox for phage genome editing.

Here, we utilize the *Bergeyella zoohelcum* Cas13b system to establish a genetic manipulation technique for phage T4. Using a dual host system, first to introduce the desired edit and then latter to select the recombinant phage, we were able to perform gene deletions (T4 glucosyl transferases), point mutations (within *gp43*, the T4 DNA polymerase gene) and gene insertion (of Yellow Fluorescent Protein gene *yfp*). Notably, we could obtain a highly deleterious mutation, thanks to efficient counterselection of wild-type phage. The protocol was optimized to obtain edited phages within 6 h following the initial infection. We present here an encompassing view of Cas13b based genome editing, including its limitations, as well as the new isogenic T4 mutants obtained with this technique.

## Results

### Unmarked deletion of T4 *alpha*- and *beta*-*glucosyltransferases*

In contrast to the DNA-dependent targeting characteristic of the Class 2 CRISPR-Cas types II (Cas9) and V (Cpf1), the type VI (Cas13) system recognizes and cleaves RNA. Despite requiring target transcription, we reasoned that the Cas13 effector activity would be more uniform across different targets on T4 due to its non-dependence on base modifications of the transcribing protospacer containing DNA.

**FIGURE 1:**
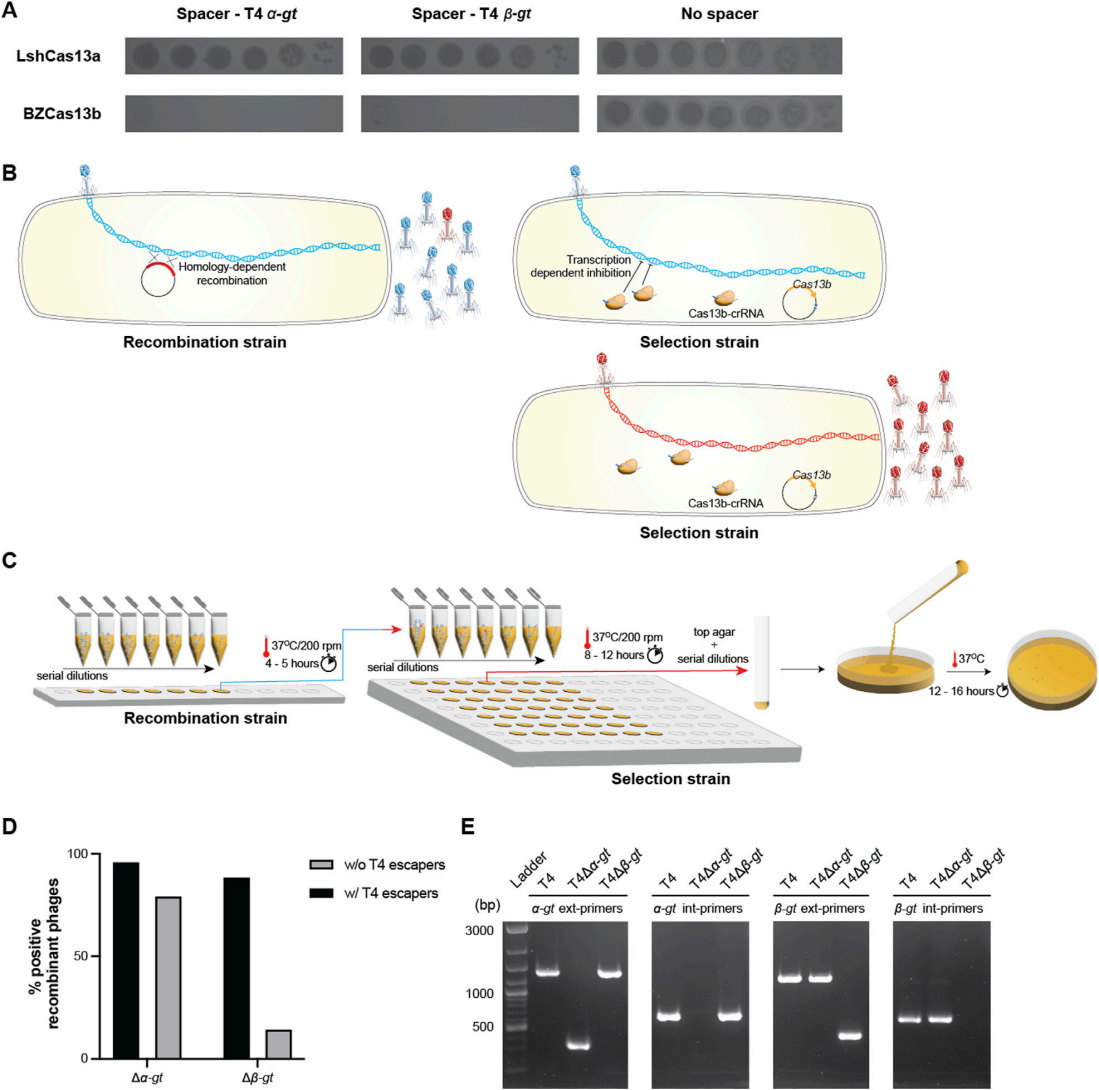
Cas13b based deletion of T4 alpha- and beta-glucosyltransferase genes. **(A)** Spot assay of 10-fold serial T4 dilutions, comparing its infectivity in *E. coli* strains expressing either plasmid-borne *Leptotrichia shahii* Cas13a (Top panel) or *Bergeyella zoohelcum* Cas13b (bottom panel), in the presence of corresponding spacers targeting either T4 *α-gt* and *β-gt,* or in the absence of spacer. **(B)** Illustration of the two-step genome editing method used. In a first step, a recombination strain carrying a plasmid containing the homologous arms necessary for deletion of the target gene was used to propagate wild-type T4. Subsequently, the resulting phage lysates were used to infect a selection strain, carrying a CRISPR-Cas13b plasmid along with a spacer specific to the transcript of the target gene, to purify the recombinants. **(C)** Illustration of the practical details of the methodology. Both phage amplification steps were realized in 200 μL cultures, at different M.O.I. Cultures that showed clear signs of lysis in the first step were serially diluted and used to infect the selection strain. **(D)** Percentage of wells comprising a mixture of T4 variants including the recombinant phage (w/T4 escapers) and those with only the expected recombinant phage (w/o T4 escapers). The respective PCR confirmations are shown in Supplementary Figures S2, S3. **(E)** PCR confirmation of the purified recombinant phages T4Δ*α-gt* and T4Δ*β-gt*, using external primers, across the *α-gt* (*α-gt* ext primers) or *β-gt* (*β-gt* ext primers) and internal primers, within the *α-gt* (*α-gt* int primers) or *β-gt* (*β-gt* int primers). Ladder: 1 kb + DNA ladder. Original image–Supplementary Figure S11.

Previously, it had been shown that among the classified Cas13 systems, Cas13a and Cas13b were the most widespread subtypes, with the former dispersed among several bacterial phyla (Adler et al., 2022; Makarova et al., 2020). Accordingly, we chose to work with Cas13a (C2c2) from *Leptotrichia shahii* (Abudayyeh et al., 2016) and Cas13b from *Bergeyella zoohelcum* ATCC 43767 (Smargon et al., 2017). First, we compared the targeting efficiency of Cas13a and Cas13b against the T4 genes *alpha-glucosyltransferase* (*α-gt*) and *beta-glucosyltransferase* (*β-gt*). Cas13 subtype specific protospacers were identified and corresponding spacers were cloned into the respective plasmids pC0003 (expressing Cas13a) and pBzCas13b. A 6-log decrease in T4 plaquing ability was observed upon expression of either *α-gt* or *β-gt* specific spacers along with Cas13b as compared to Cas13a, which had no significant impact on the plaquing ability (Figure 1A). Based on these results we concluded that targeting of T4 by BzCas13b was also likely to be more effective on others targets and hence all subsequent experiments were conducted utilizing this subtype.

Type II and V CRISPR-Cas targeting introduces a dsDNA break into the target genome, which improves the homologous recombination-repair process and simultaneously selects for the recombinant phage. RNA targeting by Cas13 does not introduce any nicks in the genome and is inherently dependent on the systems encoded within the host or the phage to promote recombination, independent of any induced dsDNA breaks. To overcome this disadvantage, we adopted a two-step system (Ramirez-Chamorro et al., 2021; Adler et al., 2022; Zhang et al., 2023), the first step to introduce the desired modification and the second to select for the recombinant phage, as illustrated in Figure 1B.

For the deletion of both *α-gt* and *β-gt*, homologous arms (approx. 80 bp in length) identical to the respective 5′ end and 3′ end of the genes were fused together (forming the desired deletion) and cloned into the pBAD24 vector. *E. coli* DH10B strain carrying the resulting plasmid was infected by wild-type T4 at MOI (multiplicity of infection, i.e., the number of phage per bacteria) ranging from 1 to 10^–6^ and incubated at 37°C for 5 h (Figure 1C; Supplementary Figure S1). Subsequently, the supernatants of each culture, containing a mixture of recombinant and wild-type T4, were serially diluted and propagated in the selection strain, carrying the plasmid-borne Cas13b and a spacer targeting either *α-gt* or *β-gt*, at 37°C for 8–12 h (Figure 1C; Supplementary Figure S2). All cultures that showed cell lysis, corresponding to MOI above 10^–3^, were then screened by PCR for the presence of recombinant phage. In the case of *α-gt* deletion, most cultures contained only the recombinant phage and a small minority also contained phage escapers (Figure 1D; Supplementary Figure S2). In case of *β-gt* deletion, the same pattern was observed, though with a larger proportion of cultures containing also wild-type phage or phage escapers in addition to the phage recombinants (Figure 1D; Supplementary Figure S3). In both cases, the residual amount of wild-type phage increased with the MOI of the recombination step. Single plaques of the recombinant phages (named T4Δ*α-gt* and T4Δ*β-gt*) were isolated and the deletion verified by PCR (Figure 1E).

Together, these results show that Cas13b from *Bergeyella zoohelcum*, in the presence of a suitable spacer, can efficiently inhibit the propagation of phage T4. Furthermore, this inhibitory effect can be harnessed to modify the genome of the T4 phage, and was utilized here to construct two new T4 phage variants, T4Δ*α-gt* and T4Δ*β-gt*. The recombination frequency was estimated to be 2.2 × 10^−3^% post recombination without selection (Supplementary Figure S4). Despite the shorter homologous arms utilized and the resulting lower frequency of recombination, strong targeting by the CRISPR-Cas13b system results in selection of recombinant phage with the desired mutation over escaper phages. Sequencing of T4Δ*α-gt* and T4Δ*β-gt* genomes revealed they were isogenic to the ancestral phage, with the exception of a single point mutation in a tail protein in the former, and two point mutations in the latter, one in a tail protein and the other in a hypothetical protein (Supplementary Figure S5). Given the high T4 spontaneous mutation rate (Drake, 1991), these point mutations most probably arose during the different growth phases of the phage or may have been a small fraction of the initial population which was eventually selected for during the recombinant phage purification process.

### Substitutions in the exonuclease domain of T4 DNA polymerase

**FIGURE 2:**
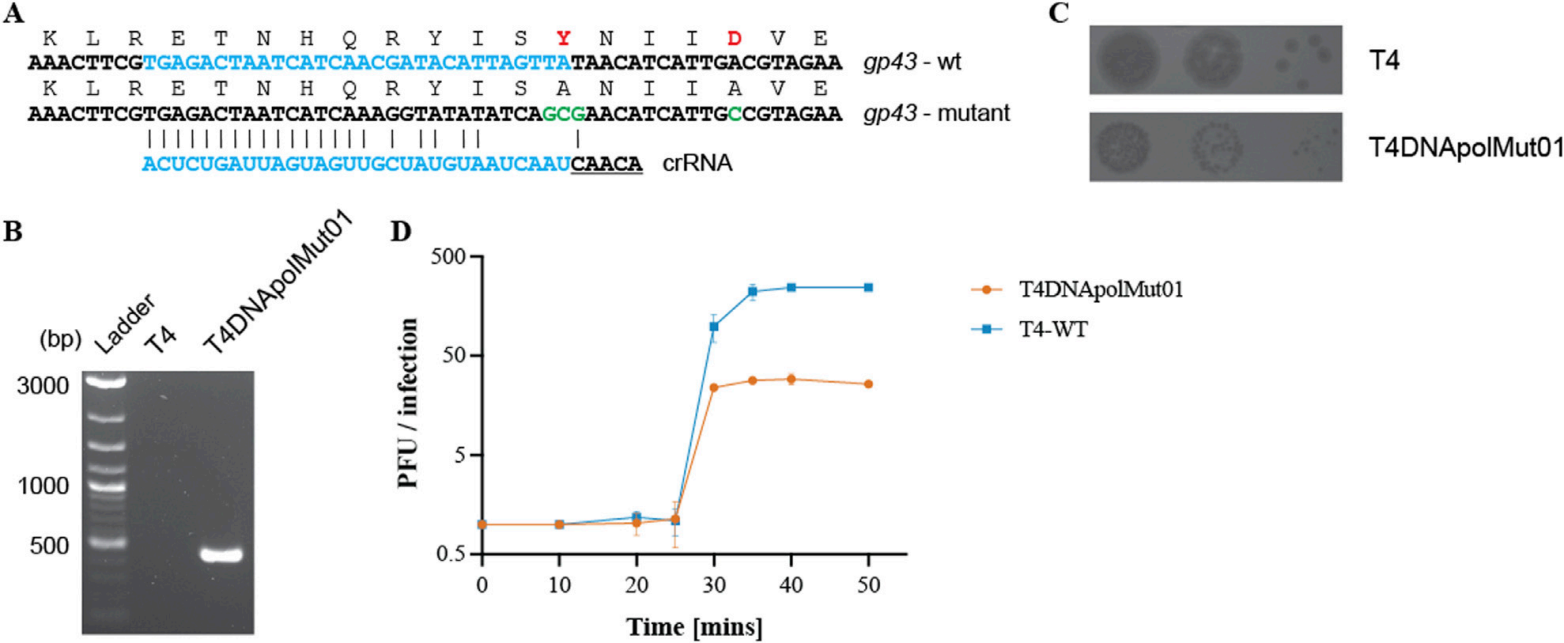
Mutation of residues in the exonuclease domain of T4 DNA polymerase. **(A)** Amino acid and nucleotide changes in *gp43* mutated gene (*gp43*_*Y320AD324*_) compared to wild-type gene, and sequence of the spacer targeting only the wild-type gp43 sequence. Additional mutations were introduced to improve mutant phage targeting escape. **(B)** PCR confirmation of the presence of the desired mutations in T4DNApolMut01, with the wild-type T4 phage used as a negative PCR control. Primer pair: T4_DNApol_chk_for and DNApolY320Amut_chk_rev. The reverse primer has a reduced binding affinity for the wild-type *gp43* gene. Original image–Supplementary Figure S12. **(C)** Spot assay of the wild-type T4 phage and the mutant phage showing differences in their plaque size. **(D)** One-step growth curve of wild-type T4 and T4DNApolMut01on *E. coli* DH10B. Data shown are the mean of three biological replicates, represented as mean value ±SD.

To further demonstrate the suitability of the type VI CRISPR-Cas system for the genetic manipulation of bacteriophages, we introduced mutations into a functionally significant phage protein domain. T4 DNA replication is processed by T4 DNA polymerase (T4 DNA pol), possessing a N-terminal proofreading exonuclease domain. Various mutants of the T4 DNA pol have been characterized, predominantly *in vitro*, including mutants in the exonuclease domain. Two amino acid residues in particular, Y320 and D324, have been shown to participate in the active site and metal ion coordination, respectively. Reduced polymerase and exonuclease activities were observed upon the substitution of amino acids Y320 and D324 by alanine, compared with the activities of the T4 wild-type polymerase enzyme (Abdus Sattar et al., 1996). To introduce these mutations, a DNA fragment of approximately 160 bps, spanning the site to be modified with nucleotide mutations necessary for the substitution of Y320 and D324 by alanine, was cloned into a plasmid vector (recombination plasmid). Additional silent mutations were also introduced into this recombination fragment to aid the recombinant phage escape targeting by Cas13b (Figure 2A) (Tambe et al., 2018; Wessels et al., 2020). Genome editing was performed as described previously; first by multiplying T4 in a strain carrying the recombination plasmid containing the polymerase gene fragment to be modified, and then by growing the mixture of wild-type and recombinant phages on a selection strain expressing *cas13b* and a spacer targeting the wild-type DNA pol (Supplementary Table S5). The mutations within the T4 DNA polymerase recombinant phage (named T4DNApolMut01) was confirmed both by PCR (Figure 2B) and Sanger sequencing (Supplementary Figure S6).

Compared to the wild-type T4 phage, the plaque size of T4DNApolMut01 was considerably smaller (Figure 2C). We estimated the burst sizes of wild-type T4 and T4DNApolMut01 to be 245 and 29, respectively, which corresponds to a 8.5-fold decrease associated with the DNA Pol mutations (Figure 2D). Despite the mutations in the exonuclease domain affecting the DNA synthesis rate and subsequently the virus yield, the latency period of the virus remained unaffected, with both wild-type and mutant phage lysing *E. coli* cells within a window of 25–30 min post infection. This mutation is therefore highly deleterious for the phage, disfavoring the growth of recombinants.

Taken together, these results show that Cas13b, with significant number of mismatches in the protospacer region, can be exploited to introduce point mutations into the T4 genome, even deleterious ones, further demonstrating that the type VI system is viable as a tool for genome editing in bacteriophages.

### Tracking T4 infection in single-cells

**FIGURE 3:**
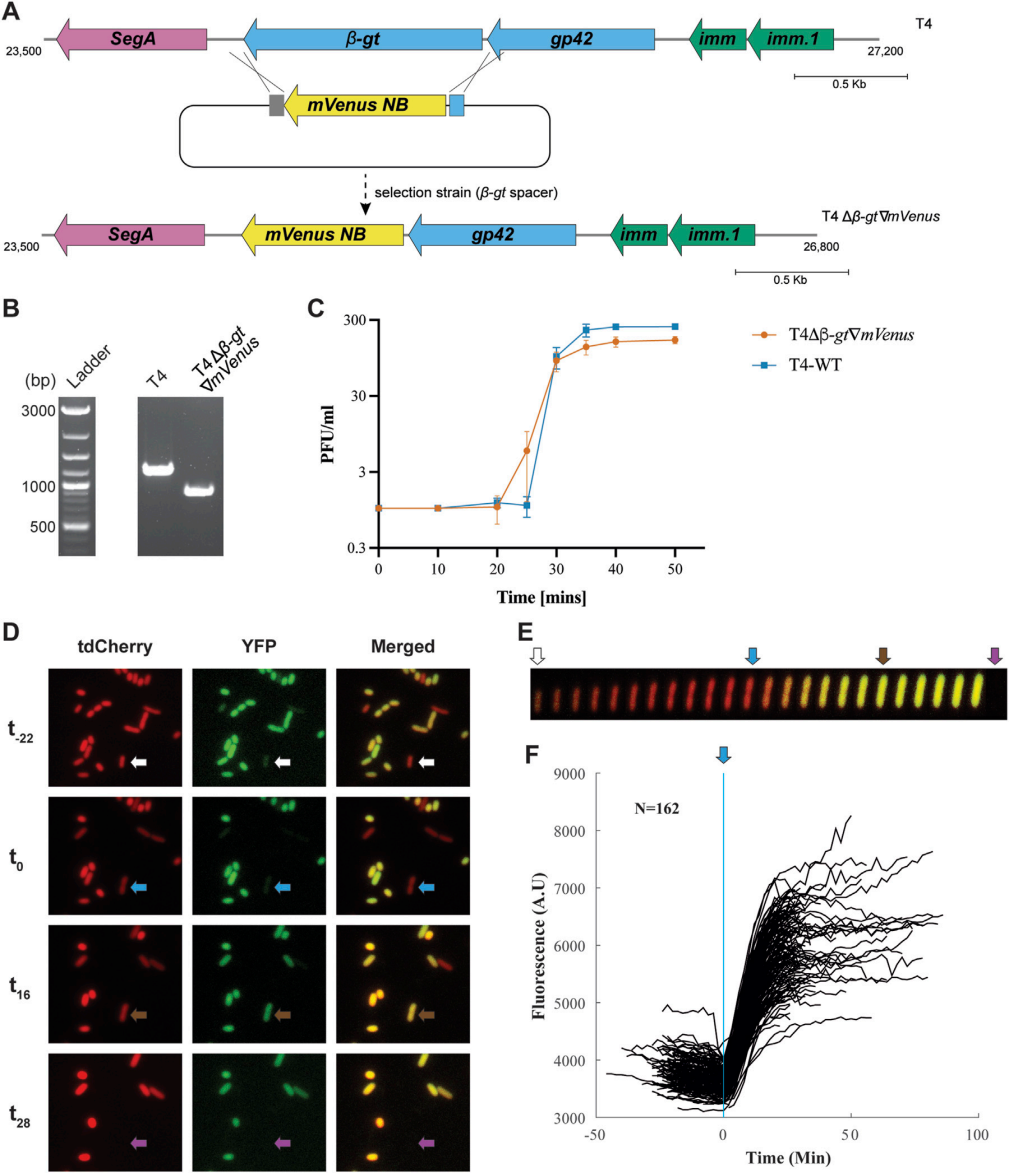
Construction and analysis of *yfp* encoding T4 phage. **(A)** Gene map of *β-gt* neighborhood in wild-type T4 (upper panel) and the intended recombination and selection (with spacer targeting the *β-gt* gene) to introduce *yfp*, replacing the *β-gt* gene. **(B)** PCR confirmation of the replacement of *β-gt* with *mVenusNB*, the wild-type T4 phage was used in the control reaction. Original image–Supplementary Figure S12. **(C)** One-step growth curve of T4Δ*β-gt*∇*mVenus*. Data shown are mean of three biological replicates, represented as mean value ±SD. **(D)** Fluorescent microscopy images of *E. coli* MG1655 PRNA1::*tdCherry* upon infection with T4Δ*β-gt*∇*mVenus* at four different times. From top to bottom: -22, 0, 16 and 28 min from start of infection of the cell identified by the arrow, with tdCherry, YFP and merged channels from left to right. Image acquisition on agar pads started approximately 20 min after the mixing of phage and bacteria (Supplementary Material S1). **(E)** Kymograph of the single cell identified in D, with tdCherry and YFP merged signals. **(F)** YFP mean fluorescent intensity evolution with time of 162 infected cells, synchronized based on the time point at which elongation stops.

Taking advantage of the editing technique established, we aimed to visualize phage infection by expressing a fluorescent protein under the control of a native T4 phage promoter. Since we aimed to visualize infection from the beginning of the lytic cycle, we choose to insert the fluorescent protein gene in T4 *β-gt*. Indeed, T4 *β-gt* is a T4 early gene, expressed within minutes after infection (Supplementary Figure S7), from the promoter of the adjacent dCMP hydroxymethylase *gp42* gene (Wolfram-Schauerte et al., 2022). For the same reason, because of its very short maturation time (*t*_50_ of 4.1 min at 37°C), we reasoned that mVenusNB (Yellow-green), would be an appropriate fluorescent protein (Balleza et al., 2018). The *mVenusNB* coding sequence was thus inserted in between 80–100 bps long arms, homologous to the upstream and downstream intergenic regions flanking the *β-gt* gene in T4 (Figure 3A), and cloned into the recombination plasmid. Additionally, the native *β-gt* SD (Shine-Dalgarno) sequence was modified into a stronger SD sequence (Supplementary Figure S8). Like before, the *E. coli* DH10B strain carrying the recombination plasmid was infected with wild-type T4 phage, following which the recombinant phages were isolated using a selection strain expressing spacers targeting *β-gt*. T4 recombinants containing *mVenusNB* coding sequence, named T4Δ*β-gt*∇*mVenus,* was confirmed by PCR on individual plaques (Figure 3B). The insertion was estimated to slightly reduce the burst size (from 245 for T4 wild-type to 155) of the T4Δ*β-gt*∇*mVenus* phage compared with wild-type T4 phage (Figure 3C).

To characterize T4Δ*β-gt*∇*mVenus* phage derived yellow fluorescence during infection, we tracked infected *E. coli* MG1655 *PRNA1::tdCherry* cells (Supplementary Table S3) by fluorescence microscopy. All *E. coli* cells express red fluorescence from the tdCherry protein, while T4 infected cells were identified by yellow fluorescence (Figures 3D, E and Supplementary Material S5). Besides the appearance of yellow fluorescence, infection is also characterized by an abrupt decrease in cell elongation and an increase in cell width (Figure 3E) enabling to estimate the time of infection. Most infected cells lyse 25–30 min after cell elongation has stopped, which corresponds to the duration of T4 life cycle as determined from the single burst assay, corroborating that elongation stop corresponds to the time of infection (Figure 3C). Some cells lyse only after 90 min, which is expected due to the lysis inhibition phenotype expressed by T4 phage. Analysis of fluorescence intensity in 162 lysing cells, synchronized based on the time at which cell elongation stops, demonstrates a sharp increase in fluorescence intensity few minutes after infection, until reaching a plateau towards the end of the phage life cycle (Figure 3F), in line with previously described β-gt protein expression (Wolfram-Schauerte et al., 2022).

Overall, we have demonstrated the possibility of gene insertion in T4 genome employing Cas13b targeting, and shown the interest of such a construct for tracking T4 infection in single cells.

### Optimizing T4 genome editing

Although the previously described Cas13 based genome editing protocol is already efficient, we aimed at reducing the time necessary to obtain phage mutants by shortening both the recombination and the selection steps. The recombination step can be shortened by infecting cells at a high MOI, as we have shown previously that despite decreasing the number of phage lytic cycles on the recombinant strain, and therefore the chances of recombination, a high MOI still enabled us to obtain phage mutants (Supplementary Figures S2, S3).

**FIGURE 4:**
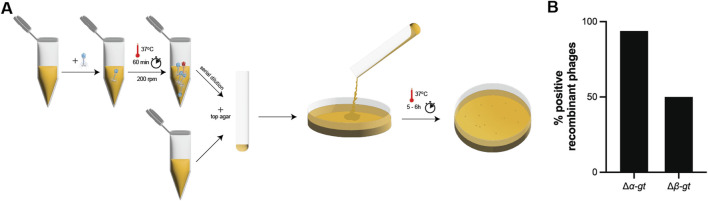
Fast T4 genome editing protocol. **(A)** Illustration of the fast genome editing method. Wild-type T4 phage is propagated on the recombination strain carrying the plasmid necessary for the homologous recombination dependent modification of the target, for 1 h at 37°C. Subsequently, the phage lysate obtained was serially diluted, and directly plated on the selection strain, carrying a CRISPR-Cas13b plasmid along with a spacer specific to the transcript of the target gene, which blocks the propagation of the wild-type phage and enable to isolate the recombinant phage. **(B)** Percentage of recombinant phages post selection with the selection strain, as shown by PCR (shown in Supplementary Figure S9).

In order to test the shortened procedure, the recombination strain for the deletion of *α-gt* was infected at an MOI of 0.1 and 1.0 and incubated at 37°C for only 60 min (instead of 5 h as demonstrated previously), which was sufficient to ensure complete host lysis. Subsequently, serial dilutions of the supernatants were directly spotted on to the selection strain, carrying the plasmid-borne Cas13b and a spacer targeting *α-gt*, without first growing the mixture on the selection strain. Plates were incubated at 37°C for 5 hours (Figure 4A). Amongst the 32 individual plaques screened by PCR, 30 corresponded to the recombinant phage (Figure 4B; Supplementary Figure S9A). This quick protocol was repeated for the deletion of *β-gt*, also with a positive outcome, as 50% of screened plaques corresponded to the recombinant phage (Figure 4B; Supplementary Figure S9B).

Upon Sanger sequencing of the escaper phages across the protospacer region, we observed either large microhomology based deletions of the protospacer region or modifications within the protospacer region, which prevented Cas13b-dependent targeting (Supplementary Figure S10). Overall, here we tested a rapid protocol to obtain different types of mutations in phage T4, and established the possibility of genome editing within 6 h.

### Variability of Cas13b crRNA efficacy in T4

**FIGURE 5:**
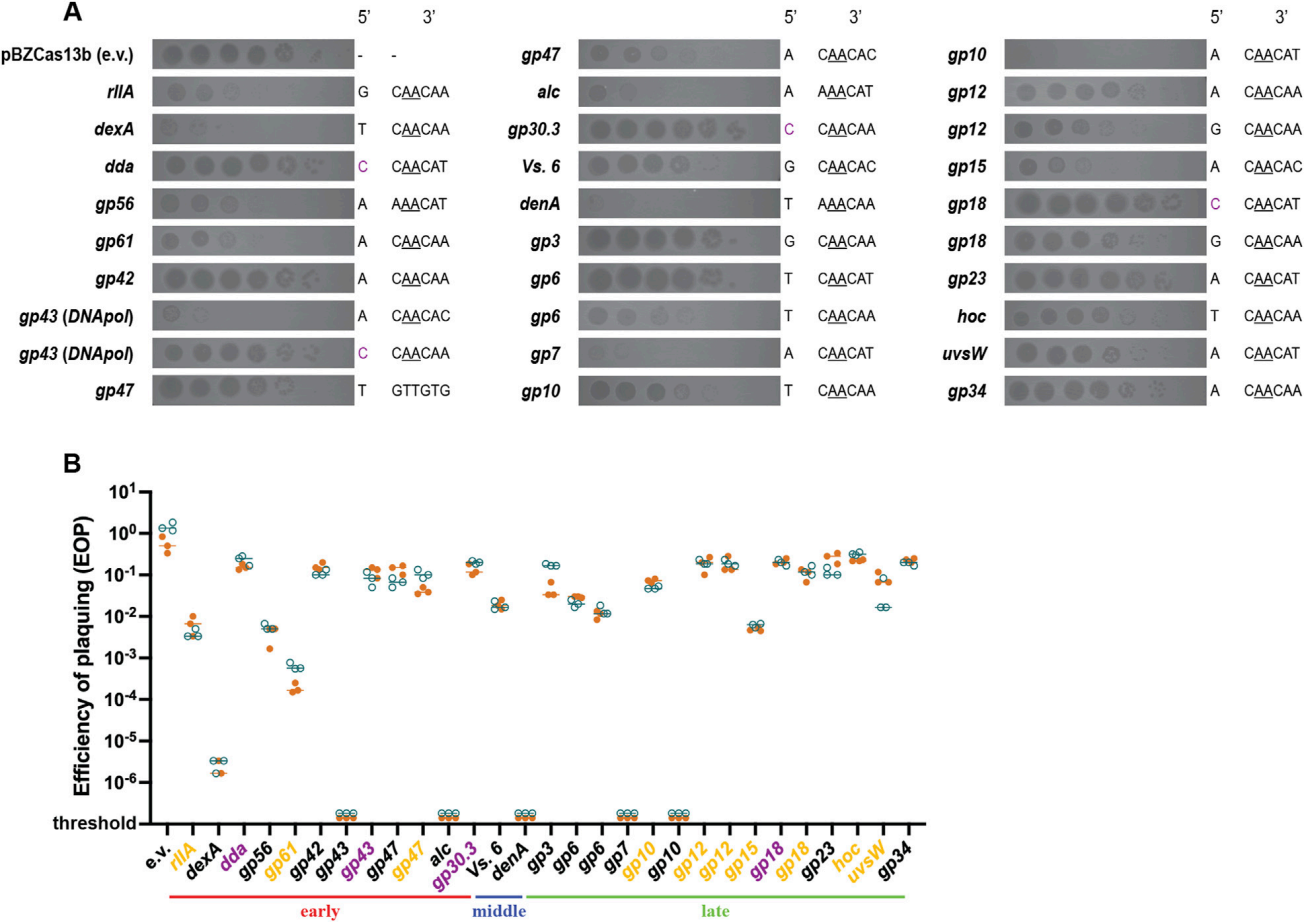
Plaquing efficiency with CRISPR-Cas13b spacers targeting different genes in phage T4. **(A)** Dilutions of wild-type T4 were spotted on *E. coli* DH10B carrying Cas13b specific spacers inserted within the pBzCas13b plasmid. Gene names of the corresponding transcripts and the 5′- and 3′- protospacer-flanking site (PFS) of the spacers are shown on either side of the respective spot assay image. Previously validated 3′ PFS motif (Smargon et al., 2017), requiring adenine at positions −2 and −3 are shown (underlined) in all protospacers containing this motif. **(B)** Quantification of the plaquing efficiency of T4 phage on different Cas13b spacer carrying strains of DH10B. Values shown are technical triplicates of two biological duplicates (shown in orange and deep teal). In some cases, the number of plaque forming units could not be estimated (shown as below threshold values). The target T4 genes (shown in X-axis) are grouped according to their expression timing during lytic cycle into early, middle and late genes. The classification was based on a previous transcriptomic study (Wolfram-Schauerte et al., 2022). Genes highlighted in yellow represent Cas13b spacer targets resulting in significantly smaller plaques than in the absence of targeting, as shown in **(A)**, regardless of the EOP value. Genes highlighted in purple represent protospacers with 5′ cytosine PFS.

In order to better delineate the Cas13b targeting efficiency against different T4 genes, we introduced in the Cas13b encoding plasmid 29 spacers complementary to protospacers situated on 23 different T4 genes (Supplementary Table S1). As efficacy could depend on the timing and strength of transcription, we choose early genes (expressed during the first 5 minutes following infection), middle genes (expressed between 5 and 10 min) and late genes (expressed after 10 min). The trans-cleavage activity of the type VI system makes targeting efficiency independent of the essentiality of the gene in the T4 life cycle. The protospacers were chosen based on the likelihood that the flanking sequence (PFS - Protospacer Flanking Sequences) would be suited for targeting by the Cas13b system (Meeske and Marraffini, 2018), NAA sequence downstream of the protospacer along with maximal non-complementarity between the anti-tag and 5′-tag sequence of the spacer to reduce any anti-tag based inhibition of targeting (Smargon et al., 2017; Wang et al., 2021). Few spacers that do not follow these rules were also tested. To evaluate spacer targeting efficiency, we looked at the reduction of PFU upon plating on strains expressing each spacer. As seen through the spot assay of serial T4 dilutions, targeting by Cas13b varied from highly efficient to no efficiency (Figure 5). Some spacers resulted in a more than 5-log decrease in plaquing efficiency compared to plaquing without selection (*denA*, *gp43*, *dexA*, *gp7*, *gp10*), others had intermediate level of targeting, with 1-log to 4-log decrease in plaque formation (*alc*, *gp56*, *res*, *gp61*, *gp6* (x2), *Vs. 6*, *gp15*, *gp10*, *hoc*, *rIIA*, *gp18*, *gp12* (x2) and *uvsW*), and finally some spacers showed no targeting at all (*gp43*, *res*, *gp3*, *gp34*, *gp18*, *gp42*, *dda*, *gp30.3*, *gp23*) (Figure 5).

Among the 29 protospacers analyzed 28 contained the recommended 3′PFS and 24 protospacers followed both the 5′ and 3′ PFS recommendations (Smargon et al., 2017). Despite this, targeting was not uniform across all these 24 target mRNAs. No clear pattern of transcription could be related to targeting efficiency. In addition, protospacer sequence-dependent differences in Cas13b-targeting were observed among single transcripts of *gp43*, *res*, *gp6*, *gp10*, *gp18* and *gp12*, reinforcing the idea that the differences do not exclusively depend on the gene transcription pattern.

## Discussion

DNA hypermodification is a frequent feature in virulent phages (Hutinet et al., 2021), a phenomenon that most likely results from the coevolutionary arms race between phages and bacteria, as it enables phages to escape most restriction enzymes. DNA hypermodification also constrains the cross-target consistency of type II and type V CRISPR-Cas activity, in turn influencing the efficiency of genome editing in hypermodified phages (Vlot et al., 2018; Bryson et al., 2015; Liu et al., 2020). These properties probably explain why some hypermodified phages, such as phages from the *Tevenvirinae* subfamily (or T4 like phages) have a broader than average host spectrum (Mathieu et al., 2020), making them attractive for phage therapy against Gram-negative bacteria (Pirnay et al., 2024) Development of genetic tools to modify these phages is therefore particularly important, both to characterize the details of phage-bacteria interactions and to engineer optimized phages for broader use against pathogenic bacteria.

Here, we report a CRISPR-Cas type VI-B based tool that can be utilized in T4 for the selection of recombinant phage post genome editing, recombinants being obtained thanks to the inherent phage or bacterial recombination machineries. In addition to the recent studies on phage genome editing using the type VI-A system, this study presents a crucial extension to the hypermodified phage genome editing toolbox.

Thanks to this technique, we obtained several significant T4 mutants of interest (Figure 1). In the mid 1960s, glucosyltransferase mutants of T2, T4 and T6 phages (referred to as T2gt, T4gt and T6gt) were obtained based on non-specific chemical mutagenesis techniques and isolated as “host-defective” mutants (Revel et al., 1965; Georgopoulos, 1967), and these mutants are still used today to study the impact of cytosine glucosylation on type II and type V CRISPR-Cas system targeting (Yaung et al., 2014; Bryson et al., 2015). The introduction of mutations outside the region of interest, as well as the possibility of reversion, are two main drawbacks of this classical mutagenesis method, that might compromise some results. The precise deletions of entire open reading frames obtained in our work guarantee that the phenotype observed cannot be related to other unintended mutations, except few point mutations that inevitably arise during phage growth and were selected during the purification process for the recombinant phage. Our short editing procedure, by minimizing the number of phage generations, should therefore also reduce the risk of introducing such mutations. In addition, by inserting a YFP fluorescent reporter within T4 genome, we were able to visualize individual infections by phage T4 (Figure 3). Potential applications include an accurate analysis of phage T4 life cycle under different conditions, as well as the determination of the expression pattern of individual T4 genes, thereby providing a high-resolution alternative to RNA sequencing. Finally, introduction of point mutations that diminish the polymerase activity of the T4 DNA Pol demonstrated the possibility to obtain highly deleterious mutations using the BzCas13b system (Figure2).

The two previously published studies on phage Cas13a targeting have reported contrasting results concerning the variability of efficiency across different protospacer regions (Guan et al., 2022; Adler et al., 2022). The targeting of phage T4 using LbuCas13a was shown to have near similar efficiencies across different protospacers, despite them being located on phage transcripts with differing expression levels and patterns. In contrast, considerable variability in the targeting of phiKZ using LseCas13a was observed, both with protospacers corresponding to different transcripts and within the same transcript. The former study also reported lower efficiency and variability using the RfxCas13d system. In our study, we observed a 6-log lower targeting efficiency with the LshCas13a system in comparison with the BzCas13b, but our comparison was limited to spacers targeting the *α-gt* and *β-gt* glucosyl transferase genes (Figure 1A). Using the BzCas13b system, we observed important variability across different protospacers, similarly to that observed with LseCas13a in targeting of phiKZ, despite using the recommended 5′ and 3′ PFS (Smargon et al., 2017). We observed that the presence of cytosine at the 5′ PFS end of the protospacer leads to inhibition of targeting (Figure 5), in line with previous observations with BzCas13b. Differences in expression timing and levels of the protospacer containing transcript, as well as potential formation of RNA secondary structures encompassing the target sequences are possible explanations for the differences seen among protospacers of different genes and between protospacers targeting the same gene (Kuo et al., 2024; Kimchi et al., 2023). The complexity of factors that determine targeting efficiency and the need for transcription are perhaps the major drawbacks of RNA based genome editing systems. Screening several protospacers for the same target and utilization of multiple spacers in parallel should potentially help in obtaining consistent genome editing outcomes via an enhancing of targeting efficiency.

Finally, we found that Cas13b mediated T4 genome modification can be achieved very rapidly. First, the high efficiency of the Cas13b based selection allowed us to perform gene deletions and insertion, as well as point mutations, with edit flanking homologous arms under 100 bps long, which greatly facilitates the construction of the plasmids for recombination. Previous studies have utilized individual flanking sequences in length greater than 250 bps for editing, exceptionally around 50 bps flanking sequence were utilized in a single case for the introduction of mutations (Adler et al., 2022). Second, despite the short edit-flanking homologous arms utilized, only 1 to 2 cycles of phage propagation were necessary to obtain sufficient recombinant phages. Third, we found that direct plating on the Cas13b and crRNA expressing strain was sufficient to select the recombinants (Figure 4). Taken together, these last two points enabled the development of a 6-hour protocol for genome editing.

In conclusion, this study presents a comprehensive view of the possibility of utilization of BzCas13b for genome engineering of T4 like bacteriophages and other phages with heavily modified DNA, given its independency of DNA modifications, complementing recent studies utilizing the type VI-A system for genome editing of bacteriophages.

## Materials and methods

### PCR, plasmids, strains and growth conditions

*Escherichia coli* DH10B (Supplementary Table S3) was utilized for all experiments including genome editing and plaque assays. All *E. coli* cultures were grown in Luria-Bertani (LB) medium, incubated at 37°C with shaking at 200 rpm. Oligonucleotides containing sequences corresponding to the homologous regions for T4 genome deletion and mutation were fused together by Overlap Extension-PCR and cloned into the plasmid pBAD24 via Circular Polymerase Extension Cloning (Quan and Tian, 2011). In case of *yfp* insertion into the genome of T4, oligonucleotides with the homologous regions were used to amplify the *mVenusNB* gene. The amplified product was cloned into the pBAD24 plasmid via Circular Polymerase Extension Cloning. Bacteriophage targeting spacers were introduced into plasmids pC0003 (#79152), containing the Cas13a locus from *Leptotrichia Shahii*, and pBzCas13b (#89898), encoding the Cas13b from *B. zoohelcum* ATCC 43767, both obtained from Addgene. *mVenusNB* gene was derived from pEB1-mVenusNB, obtained from Addgene (plasmid #103986). Overlapping oligonucleotides with 5′ and 3′ extensions were mixed together at a final concentration of 1 μM with PCR reaction buffer, incubated at 95°C for 10 min and subsequently slow cooled to facilitate annealing. T4 targeting plasmids were constructed by ligating BsaI digested plasmids with the annealed spacers. All primers utilized in this study are listed in Supplementary Table S2. All polymerase chain reactions (PCRs) were performed with the respective primers using the Phusion High-Fidelity DNA Polymerase (NEB).

### Phage preparation, plaque assay, spot assay and EOP (efficiency of plating)

To obtain phage stocks, *E. coli* DH10B culture at an OD_600_ of 0.2 was infected with phage T4 at a MOI comprised between 0.01 and 0.1, and grown at 37°C with shaking until complete lysis was observed. After centrifugation, at 6,300 x g for 5 min, to pellet the bacterial cells, supernatants containing phage were filtered using a 0.2 µm sterile filter to eliminate remaining cells.

For plaque assay, 100 μL overnight bacterial culture was mixed with preheated top agar (0.4% agar) along with the respective phage dilution and poured onto LB agar plates (containing the appropriate antibiotics, when necessary). The plates were incubated overnight at 37°C unless otherwise specified. For spot assay, 3–5 μL of phage dilutions were spotted onto a solidified layer of 3 mL of top agar containing 100 μL of overnight bacterial culture. EOP were estimated by spot assay, by spotting serial dilutions of the T4 wild-type onto top agar mixture containing *E. coli* DH10B culture with the respective Cas13-spacer plasmid. EOP were obtained by dividing the concentration of T4 plaque forming units (PFU) on the strain of interest by the PFU concentration on DH10B.

### Genome editing - long protocol

Overnight culture of the *E. coli* DH10B recombination strain was diluted 1000-fold and incubated until an OD_600_ of 0.2 was reached. Serial dilutions of phage T4 were prepared (from 1 to 10^–6^), and 10 μL of each dilution were mixed with 190 μL of the recombination strain in 96-well microtiter plates and incubated at 37°C until complete lysis was observed at all dilutions, approximately between 4 and 5 h. In the second step, similarly, overnight culture of the selection strain was diluted 1000-fold and incubated until an OD_600_ of 0.2 was reached. Supernatants obtained previously with recombination strain were serially diluted, and 10 μL of each dilution were mixed with 190 μL of the selection strain in 96-well microtiter plates and incubated at 37°C for 8–12 h. Wells with clear growth retardation were screened for positive phage recombinants with PCR. Single plaques of the recombinant phage were then obtained following plaque assay on the selection strain.

### Genome editing - short protocol

Exponentially growing *E. coli* DH10B recombination strain at OD_600_ of 0.2, prepared by 100-fold dilution of overnight culture, was infected with T4 phage at an MOI of 0.1 and incubated at 37°C for 60 min. Serial dilutions of the supernatant were spotted on the selection strain (as described earlier) and incubated at 37°C for 5 hours. Single plaques were screened for the appropriate mutation by PCR.

### One-step growth curve

Phage burst sizes were measured as described earlier (De Paepe et al., 2014). Briefly, 10 μL of phage (10^7^ PFU/mL) were mixed with 100 μL of *E. coli* DH10B, concentrated from 1 mL of exponentially growing culture (at OD_600_ = 0.2) in LB broth. The mixture was incubated at 37°C for 5 min, centrifuged, washed once with preheated LB. Infected cells were diluted 100-fold and 10,000-fold with preheated LB and incubated at 37°C. Samples were withdrawn at the appropriate time points and phage titers estimated by plaque assay.

### DNA extraction and sequencing

Preparations of the wild-type and mutant phage DNA were performed as described earlier. Phages were precipitated overnight using PEG (10%, final concentration) and NaCl (0.5 M, final concentration), centrifuged at 12,000 rpm for 30 min and resuspended in SM buffer (50 mM Tris-HCl pH 7.5, 100 mM NaCl, 8 mM MgSO4). Standard Phenol-Chloroform extraction, followed by ethanol precipitation was performed to extract total phage DNA. DNA sequencing was performed by Next-Generation Sequencing (INVIEW virus, Eurofins Genomics, Ebersberg, Germany). The sequencing reads were assembled using SPAdes (Prjibelski et al., 2020) version 3.15.3 with default parameters. Mutations in T4 genomes were identified with breseq (Deatherage and Barrick, 2014) version 0.36.1, using default parameters with the wild-type T4 genome as the reference sequence.

### Fluorescent *E. coli* strain construction, microscopy and Image analysis

MG1655 PRNA1::*tdCherry E. coli* strain was constructed using plasmid pNDL-32 obtained from Johan Paulsson’s lab (http://openwetware.org/wiki/Paulsson:Strains, as described before (Robert et al., 2018), Supplementary Table S3). Cells were grown up to an OD_600_ of 0.5 in LB at 37°C, from a 200-fold dilution of overnight culture, and T4 phage was added at an MOI of 1. Immediately after, the cell and phage mixture was spread on 1% agarose + LB pad in a microscope slide, using a Thermo Scientific™ Gene Frame. Image acquisition started ∼20–25 min after, just as the first cell lysis occurred. We used an inverted Nikon Eclipse Ti-E microscope equipped with a KINETI sCMOS camera, a CoolLED pE-800 light source with LED wavelengths at 580 nm for tdCherry and 500 nm for YFP, a Plan APO ×100 oil immersion objective (N.A. 1.45), and an Okolab temperature-controlled chamber regulated at 37°C. YFP and tdCherry exposure time and intensity were 500 ms, 5% and 200 ms, 2% respectively. Images were taken every 2 min for 2 h and image analysis was performed with OUFTI^©^ software (Paintdakhi et al., 2016), using the cytoplasmic tdCherry signal for cell segmentation. Mean pixel intensity from YFP channel, for the selected lysing cells, were extracted and plotted using Matlab Mathworks^©^ suite.

## Data Availability

The nucleotide sequences of the T4 mutant phages, T4 α-gt and β-gt, have been deposited in the European Nucleotide Archive under the accession numbers OZ209023 and OZ209057. The raw sequencing reads have been deposited, and are available at https://doi.org/10.57745/0K41WT.

## References

[B1] Abdus Sattar A. K. Lin T. C. Jones C. Konigsberg W. H. (1996). Functional consequences and exonuclease kinetic parameters of point mutations in bacteriophage T4 DNA polymerase. Biochemistry 35 (51), 16621–16629. 10.1021/bi961552q 8987997

[B2] Abudayyeh O. O. Gootenberg J. S. Konermann S. Joung J. Slaymaker I. M. Cox D. B. (2016). C2c2 is a single-component programmable RNA-guided RNA-targeting CRISPR effector. Science 353 (6299), aaf5573. 10.1126/science.aaf5573 27256883 PMC5127784

[B3] Adler B. A. Hessler T. Cress B. F. Lahiri A. Mutalik V. K. Barrangou R. (2022). Broad-spectrum CRISPR-Cas13a enables efficient phage genome editing. Nat. Microbiol. 7 (12), 1967–1979. 10.1038/s41564-022-01258-x 36316451 PMC9712115

[B4] Balleza E. Kim J. M. Cluzel P. (2018). Systematic characterization of maturation time of fluorescent proteins in living cells. Nat. Methods 15 (1), 47–51. 10.1038/nmeth.4509 29320486 PMC5765880

[B5] Bryson A. L. Hwang Y. Sherrill-Mix S. Wu G. D. Lewis J. D. Black L. (2015). Covalent modification of bacteriophage T4 DNA inhibits CRISPR-cas9. mBio 6 (3), e00648. 10.1128/mBio.00648-15 26081634 PMC4471564

[B6] Deatherage D. E. Barrick J. E. (2014). Identification of mutations in laboratory-evolved microbes from next-generation sequencing data using breseq. Methods Mol. Biol. 1151, 165–188. 10.1007/978-1-4939-0554-6_12 24838886 PMC4239701

[B7] De Paepe M. Hutinet G. Son O. Amarir-Bouhram J. Schbath S. Petit M. A. (2014). Temperate phages acquire DNA from defective prophages by relaxed homologous recombination: the role of Rad52-like recombinases. PLoS Genet. 10 (3), e1004181. 10.1371/journal.pgen.1004181 24603854 PMC3945230

[B8] Dong J. Chen C. Liu Y. Zhu J. Li M. Rao V. B. (2021). Engineering T4 bacteriophage for *in vivo* display by type V CRISPR-cas genome editing. ACS Synth. Biol. 10 (10), 2639–2648. 10.1021/acssynbio.1c00251 34546037 PMC12867177

[B9] Drake J. W. (1991). A constant rate of spontaneous mutation in DNA-based microbes. Proc. Natl. Acad. Sci. U. S. A. 88 (16), 7160–7164. 10.1073/pnas.88.16.7160 1831267 PMC52253

[B10] Duong M. M. Carmody C. M. Ma Q. Peters J. E. Nugen S. R. (2020a). Optimization of T4 phage engineering via CRISPR/Cas9. Sci. Rep. 10 (1), 18229. 10.1038/s41598-020-75426-6 33106580 PMC7588440

[B11] Duong M. M. Carmody C. M. Ma Q. Peters J. E. Nugen S. R. (2020b). Optimization of T4 phage engineering via CRISPR/Cas9. Sci. Rep. 10 (1), 18229. 10.1038/s41598-020-75426-6 33106580 PMC7588440

[B12] Edgar R. Lielausis I. (1964). Temperature-sensitive mutants of bacteriophage T4D: their isolation and genetic characterization. Genetics 49 (4), 649–662. 10.1093/genetics/49.4.649 14156925 PMC1210603

[B13] Edgar R. S. Denhardt G. H. Epstein R. H. (1964). A comparative genetic study of conditional lethal mutations of bacteriophage T4d. Genetics 49 (4), 635–648. 10.1093/genetics/49.4.635 14156924 PMC1210602

[B14] Epstein R. Bolle A. Steinberg C. M. Kellenberger E. De La Tour E. B. Chevalley R. Physiological studies of conditional lethal mutants of bacteriophage T4D. In Cold spring harb. Symp. Quant. Biol., cold spring harbor symposia on quantitative Biology, 1963; Cold Spring Harbor Laboratory Press: 28, 375–394 . Available at: https://symposium.cshlp.org/content/28/375.short . 10.1101/sqb.1963.028.01.053

[B15] Georgopoulos C. P. (1967). Isolation and preliminary characterization of T4 mutants with nonglucosylated DNA. Biochem. Biophys. Res. Commun. 28 (2), 179–184. 10.1016/0006-291x(67)90426-3 5340730

[B16] Guan J. Oromi-Bosch A. Mendoza S. D. Karambelkar S. Berry J. D. Bondy-Denomy J. (2022). Bacteriophage genome engineering with CRISPR-Cas13a. Nat. Microbiol. 7 (12), 1956–1966. 10.1038/s41564-022-01243-4 36316452 PMC9722621

[B17] Hattman S. Fukasawa T. (1963). Host-induced modification of T-even phages due to defective glucosylation of their DNA. Proc. Natl. Acad. Sci. U. S. A. 50 (2), 297–300. 10.1073/pnas.50.2.297 14060647 PMC221171

[B18] Hutinet G. Lee Y. J. de Crecy-Lagard V. Weigele P. R. (2021). Hypermodified DNA in viruses of E. coli and Salmonella. EcoSal Plus 9 (2), eESP00282019. 10.1128/ecosalplus.ESP-0028-2019 34910575 PMC11163837

[B19] Isaev A. Andriianov A. Znobishcheva E. Zorin E. Morozova N. Severinov K. (2022). Editing of phage genomes - recombineering-assisted SpCas9 modification of model coliphages T7, T5, and T3. Mol. Biol. Mosk. 56 (6), 801–815. 10.1134/s0026893322060073 36475474

[B20] Ishino Y. Krupovic M. Forterre P. (2018). History of CRISPR-cas from encounter with a mysterious repeated sequence to genome editing technology. J. Bacteriol. 200 (7), e00580–17. 10.1128/JB.00580-17 29358495 PMC5847661

[B21] Kimchi O. Larsen B. B. Dunkley O. R. S. Te Velthuis A. J. W. Myhrvold C. (2023). RNA structure modulates Cas13 activity and enables mismatch detection. bioRxiv, 10.1101/2023.10.05.560533 PMC12584110 41131153

[B22] Kiro R. Shitrit D. Qimron U. (2014). Efficient engineering of a bacteriophage genome using the type I-E CRISPR-Cas system. RNA Biol. 11 (1), 42–44. 10.4161/rna.27766 24457913 PMC3929423

[B23] Kuo H. C. Prupes J. Chou C. W. Finkelstein I. J. (2024). Massively parallel profiling of RNA-targeting CRISPR-Cas13d. Nat. Commun. 15 (1), 498. 10.1038/s41467-024-44738-w 38216559 PMC10786891

[B24] Kutter E. M. Bradley D. Schenck R. Guttman B. S. Laiken R. (1981). Bacteriophage T4 alc gene product: general inhibitor of transcription from cytosine-containing DNA. J. Virol. 40 (3), 822–829. 10.1128/JVI.40.3.822-829.1981 7321103 PMC256693

[B25] Latarjet R. (1949). Mutation induced in a virus by ultraviolet irradiation of infected cells. C R. Hebd. Seances Acad. Sci. 228 (16), 1354–1357. 18131489

[B26] Latarjet R. (1954). Induction of mutations in a bacteriophage. Acta Unio Int. Contra Cancrum 10 (2), 136. 13197161

[B27] Liu Y. Dai L. Dong J. Chen C. Zhu J. Rao V. B. (2020). Covalent modifications of the bacteriophage genome confer a degree of resistance to bacterial CRISPR systems. J. Virol. 94 (23), e01630–20. 10.1128/JVI.01630-20 PMC7654273 32938767

[B28] Makarova K. S. Wolf Y. I. Alkhnbashi O. S. Costa F. Shah S. A. Saunders S. J. (2015). An updated evolutionary classification of CRISPR-Cas systems. Nat. Rev. Microbiol. 13 (11), 722–736. 10.1038/nrmicro3569 26411297 PMC5426118

[B29] Makarova K. S. Wolf Y. I. Iranzo J. Shmakov S. A. Alkhnbashi O. S. Brouns S. J. J. (2020). Evolutionary classification of CRISPR-Cas systems: a burst of class 2 and derived variants. Nat. Rev. Microbiol. 18 (2), 67–83. 10.1038/s41579-019-0299-x 31857715 PMC8905525

[B30] Mathieu A. Dion M. Deng L. Tremblay D. Moncaut E. Shah S. A. (2020). Virulent coliphages in 1-year-old children fecal samples are fewer, but more infectious than temperate coliphages. Nat. Commun. 11 (1), 378. 10.1038/s41467-019-14042-z 31953385 PMC6969025

[B31] Mattson T. Van Houwe G. Bolle A. Selzer G. Epstein R. (1977). Genetic identification of cloned fragments of bacteriophage T4 DNA and complementation by some clones containing early T4 genes. Mol. Gen. Genet. 154 (3), 319–326. 10.1007/BF00571289 927440

[B32] Meeske A. J. Marraffini L. A. (2018). RNA guide complementarity prevents self-targeting in type VI CRISPR systems. Mol. Cell 71 (5), 791–801. 10.1016/j.molcel.2018.07.013 30122537 PMC7955661

[B33] Meeske A. J. Nakandakari-Higa S. Marraffini L. A. (2019). Cas13-induced cellular dormancy prevents the rise of CRISPR-resistant bacteriophage. Nature 570 (7760), 241–245. 10.1038/s41586-019-1257-5 31142834 PMC6570424

[B34] O'Connell M. R. (2019). Molecular mechanisms of RNA targeting by cas13-containing type VI CRISPR-cas systems. J. Mol. Biol. 431 (1), 66–87. 10.1016/j.jmb.2018.06.029 29940185

[B35] Paintdakhi A. Parry B. Campos M. Irnov I. Elf J. Surovtsev I. (2016). Oufti: an integrated software package for high-accuracy, high-throughput quantitative microscopy analysis. Mol. Microbiol. 99 (4), 767–777. 10.1111/mmi.13264 26538279 PMC4752901

[B36] Pirnay J. P. Djebara S. Steurs G. Griselain J. Cochez C. De Soir S. (2024). Personalized bacteriophage therapy outcomes for 100 consecutive cases: a multicentre, multinational, retrospective observational study. Nat. Microbiol. 9 (6), 1434–1453. 10.1038/s41564-024-01705-x 38834776 PMC11153159

[B37] Prjibelski A. Antipov D. Meleshko D. Lapidus A. Korobeynikov A. (2020). Using SPAdes *de novo* assembler. Curr. Protoc. Bioinforma. 70 (1), e102. 10.1002/cpbi.102 32559359

[B38] Quan J. Tian J. (2011). Circular polymerase extension cloning for high-throughput cloning of complex and combinatorial DNA libraries. Nat. Protoc. 6 (2), 242–251. 10.1038/nprot.2010.181 21293463

[B39] Ramirez-Chamorro L. Boulanger P. Rossier O. (2021). Strategies for bacteriophage T5 mutagenesis: expanding the toolbox for phage genome engineering. Front. Microbiol. 12, 667332. 10.3389/fmicb.2021.667332 33981295 PMC8108384

[B40] Reha-Krantz L. J. Bessman M. J. (1981). Studies on the biochemical basis of mutation VI. Selection and characterization of a new bacteriophage T4 mutator DNA polymerase. J. Mol. Biol. 145 (4), 677–695. 10.1016/0022-2836(81)90309-0 6267293

[B41] Revel H. R. Hattman S. Luria S. E. (1965). Mutants of bacteriophages T2 and T6 defective in alpha-glucosyl transferase. Biochem. Biophys. Res. Commun. 18, 545–550. 10.1016/0006-291x(65)90788-6 14301458

[B42] Revel H. R. Luria S. E. (1970). DNA-glucosylation in T-even phage: genetic determination and role in phagehost interaction. Annu. Rev. Genet. 4 (0), 177–192. 10.1146/annurev.ge.04.120170.001141 4950809

[B43] Robert L. Ollion J. Robert J. Song X. Matic I. Elez M. (2018). Mutation dynamics and fitness effects followed in single cells. Science 359 (6381), 1283–1286. 10.1126/science.aan0797 29590079

[B44] Shen J. Zhou J. Chen G. Q. Xiu Z. L. (2018). Efficient genome engineering of a virulent Klebsiella bacteriophage using CRISPR-cas9. J. Virol. 92 (17), e00534–18. 10.1128/JVI.00534-18 29899105 PMC6096830

[B45] Shmakov S. Abudayyeh O. O. Makarova K. S. Wolf Y. I. Gootenberg J. S. Semenova E. (2015). Discovery and functional characterization of diverse class 2 CRISPR-cas systems. Mol. Cell 60 (3), 385–397. 10.1016/j.molcel.2015.10.008 26593719 PMC4660269

[B46] Smargon A. A. Cox D. B. T. Pyzocha N. K. Zheng K. Slaymaker I. M. Gootenberg J. S. (2017). Cas13b is a type VI-B CRISPR-associated RNA-guided RNase differentially regulated by accessory proteins Csx27 and Csx28. Mol. Cell 65 (4), 618–630. 10.1016/j.molcel.2016.12.023 28065598 PMC5432119

[B47] Tambe A. East-Seletsky A. Knott G. J. Doudna J. A. O'Connell M. R. (2018). RNA binding and HEPN-nuclease activation are decoupled in CRISPR-Cas13a. Cell Rep. 24 (4), 1025–1036. 10.1016/j.celrep.2018.06.105 30044970 PMC6085867

[B48] Tao P. Wu X. Tang W. C. Zhu J. Rao V. (2017). Engineering of bacteriophage T4 genome using CRISPR-Cas9. ACS Synth. Biol. 6 (10), 1952–1961. 10.1021/acssynbio.7b00179 28657724 PMC5771229

[B49] Vialetto E. Yu Y. Collins S. P. Wandera K. G. Barquist L. Beisel C. L. (2022). A target expression threshold dictates invader defense and prevents autoimmunity by CRISPR-Cas13. Cell Host Microbe 30 (8), 1151–1162.e6. 10.1016/j.chom.2022.05.013 35690065 PMC9590104

[B50] Vlot M. Houkes J. Lochs S. J. A. Swarts D. C. Zheng P. Kunne T. (2018). Bacteriophage DNA glucosylation impairs target DNA binding by type I and II but not by type V CRISPR-Cas effector complexes. Nucleic Acids Res. 46 (2), 873–885. 10.1093/nar/gkx1264 29253268 PMC5778469

[B51] Wang B. Zhang T. Yin J. Yu Y. Xu W. Ding J. (2021). Structural basis for self-cleavage prevention by tag:anti-tag pairing complementarity in type VI Cas13 CRISPR systems. Mol. Cell 81 (5), 1100–1115.e5. 10.1016/j.molcel.2020.12.033 33472057 PMC8274241

[B52] Wang J. Y. Doudna J. A. (2023). CRISPR technology: a decade of genome editing is only the beginning. Science 379 (6629), eadd8643. 10.1126/science.add8643 36656942

[B53] Wessels H. H. Mendez-Mancilla A. Guo X. Legut M. Daniloski Z. Sanjana N. E. (2020). Massively parallel Cas13 screens reveal principles for guide RNA design. Nat. Biotechnol. 38 (6), 722–727. 10.1038/s41587-020-0456-9 32518401 PMC7294996

[B54] Wolfram-Schauerte M. Pozhydaieva N. Viering M. Glatter T. Hofer K. (2022). Integrated omics reveal time-resolved insights into T4 phage infection of E. coli on proteome and transcriptome levels. Viruses 14 (11), 2502. 10.3390/v14112502 36423111 PMC9697503

[B55] Yaung S. J. Esvelt K. M. Church G. M. (2014). CRISPR/Cas9-mediated phage resistance is not impeded by the DNA modifications of phage T4. PLoS One 9 (6), e98811. 10.1371/journal.pone.0098811 24886988 PMC4041780

[B56] Zhang W. Bhoobalan-Chitty Y. Zhai X. Hui Y. Hansen L. H. Deng L. (2023). Replication protein rep provides selective advantage to viruses in the presence of CRISPR-cas immunity. CRISPR J. 6 (1), 32–42. 10.1089/crispr.2022.0037 36576859

