# Supplementary material for: Genetic manipulation of bacteriophage T4 utilizing the CRISPR-Cas13b system
Source: Front Genome Ed. 2024 Dec 19;6:1495968. doi: 10.3389/fgeed.2024.1495968 (PMC11693715; doi:10.3389/fgeed.2024.1495968)
Supplement: Supplementary file 1 [file DataSheet1.pdf]

SUPPLEMENTARY FIGURES

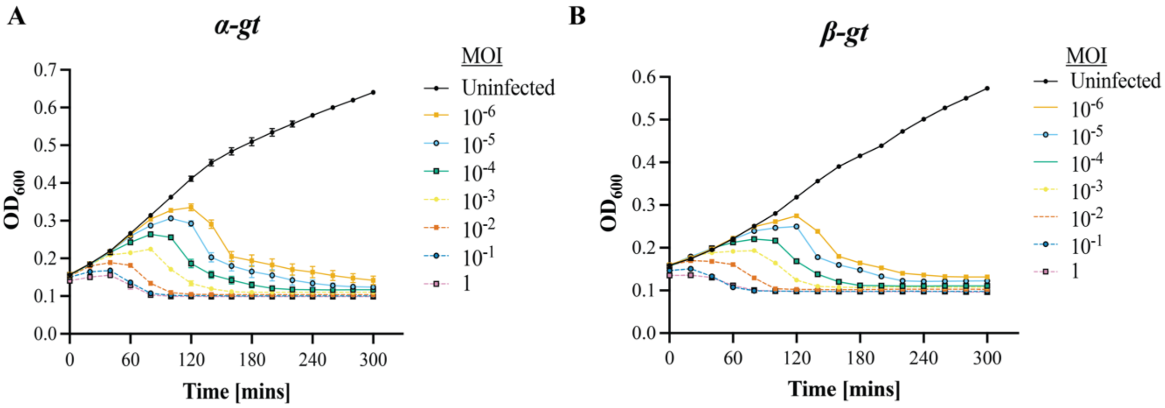

Supplementary Figure 1. Growth curves of *α-gt* and *β-gt* recombination strains infected with wild-type T4. A. Growth curve of the recombination strain carrying the plasmid for the deletion of alpha glucosyltransferase (*α-gt*) at different MOIs. **B.** Growth curve of the recombination strain carrying the plasmid for the deletion of beta glucosyltransferase (*β-gt*) at different MOIs.

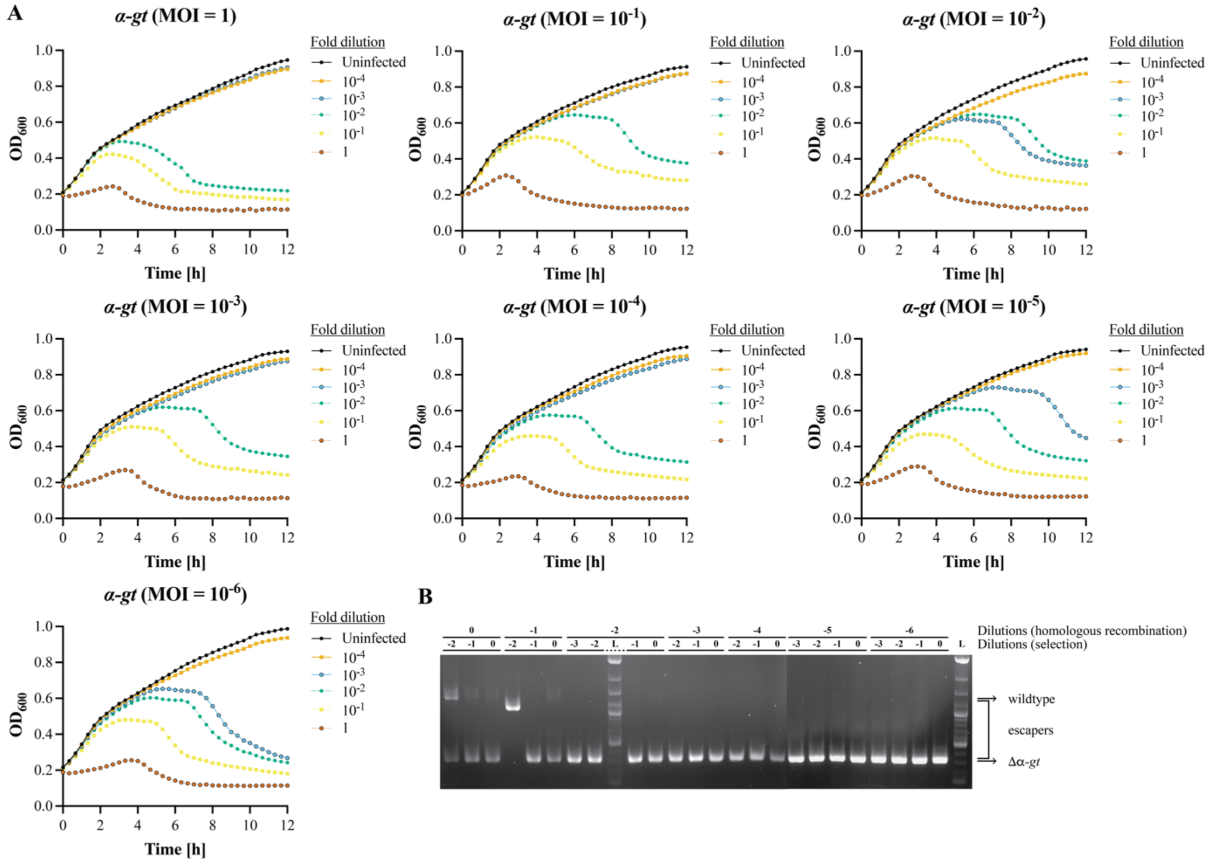

Supplementary Figure 2. Growth curves of *α-gt* selection strain infected with a mixture of recombinant and wild-type T4. A. Growth curves of the selection strain, carrying the plasmid-borne Cas13b and a spacer targeting *α-gt*, infected with serial dilutions of the phage lysates obtained from the infection of the recombination strain (Supplementary Figure 1A), containing a mixture of recombinant (Δ*α-gt*) and wild-type phages. **B.** PCR products of *α-gt* locus in phage lysates of the selection strain. Expected sizes of wild-type and deleted genes are shown by the arrows on the right. Intermediate sizes correspond to unattended deletions.

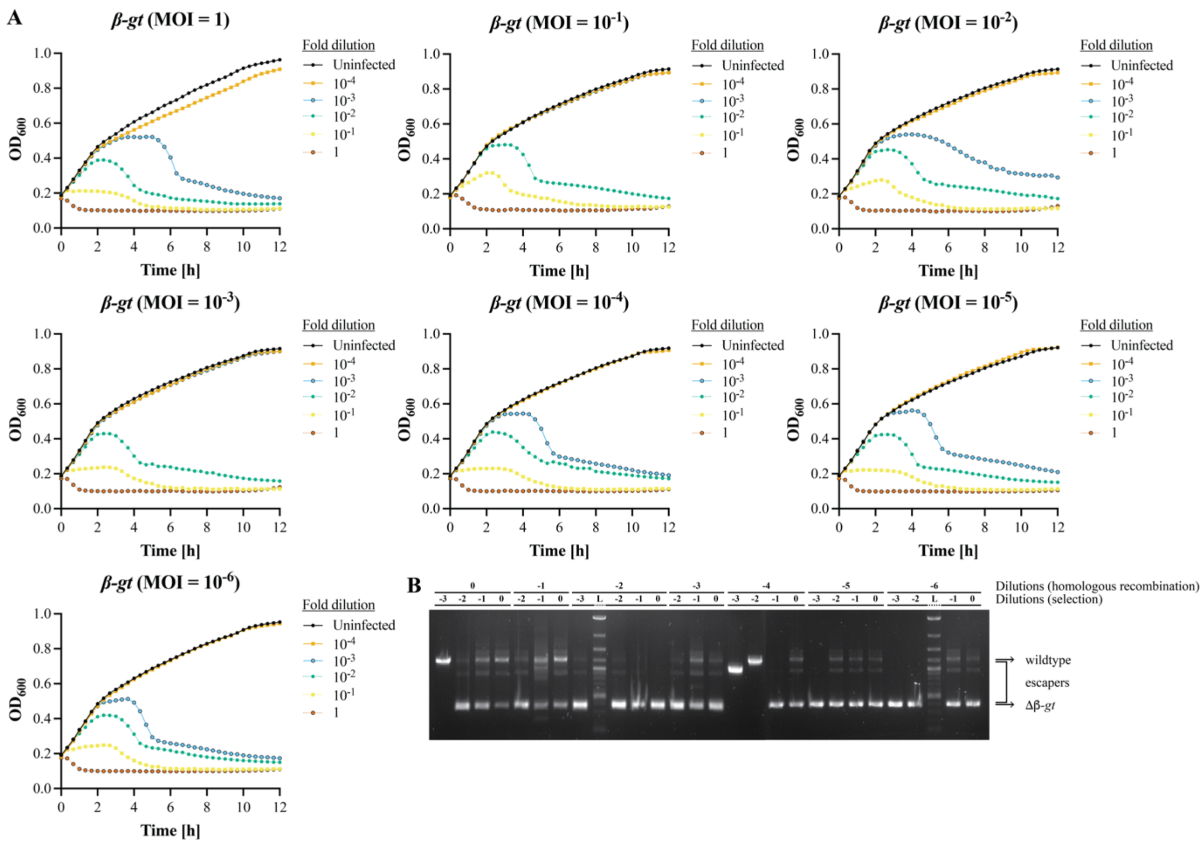

Supplementary Figure 3. Growth curves of *β*-gt selection strain infected with a mixture of recombinant and wild-type T4. Growth curves of the selection strain, carrying the plasmid-borne Cas13b and a spacer targeting *β-gt*, infected with dilutions of phage lysates obtained from infection of the *β-gt* recombination strain (Supplementary Figure 1A), containing a mixture of recombinant (Δ*β-gt*) and wild-type phages. **B.** PCR products of *β-gt* locus in phage lysates after growth on the selection strain. Expected sizes of wild-type and deleted genes are shown by the arrows on the right. Intermediate sizes correspond to unattended deletions.

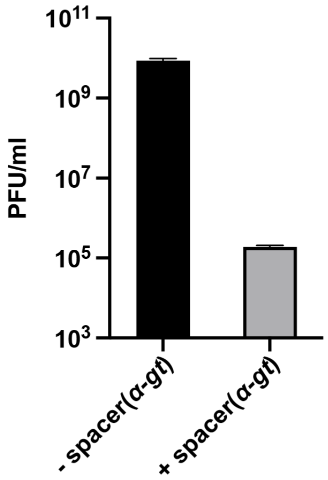

Supplementary Figure 4: Estimation of recombination efficiency. Total number of phages and the number of recombinant phages present post infection of the *α-gt* recombination strain (10^{-5}, fold phage dilution) was estimated through plaque assay with a control *E. coli* strain (lacking Cas13b targeting) and the selection strain (with Cas13b and a spacer targeting *α-gt*). Data shown are the mean of three biological replicates, represented as mean value ± SD.

breseq version 0.36.1
mutation predictions | marginal predictions | summary statistics | genome diff | command line log

Predicted mutations					
**evidence**	**position**	**mutation**	**annotation**	**gene**	**description**
MC JC	36,721	Δ1,065 bp	coding (29-1093/1203 nt)	*agt* ←	DNA alpha-glucosyltransferase
RA	159,371	C→T	T939I (ACT→ATT)	*T4_gp_00251* →	Long-tail fiber protein gp37

breseq version 0.36.1
mutation predictions | marginal predictions | summary statistics | genome diff | command line log

Predicted mutations					
**evidence**	**position**	**mutation**	**annotation**	**gene**	**description**
MC JC	24,429	Δ838 bp		*[bgt]*	*[bgt]*
RA	59,069	T→G	H42P (CAC→CCC)	*T4_gp_00102* ←	hypothetical protein
RA	84,622	A→G	N528S (AAC→AGC)	*7* →	Baseplate wedge protein gp7

Supplementary Figure 5. Mutations present in T4Δ*α-gt* (top) and T4Δ*β-gt* (bottom). Analysis of whole genome sequencing reads with Breseq computational pipeline revealed the expected deletions, as well as one (in T4Δ*α-gt*) and two (in T4Δ*β-gt*) amino acids substitutions in the recombinant phages as compared to the wild-type T4.

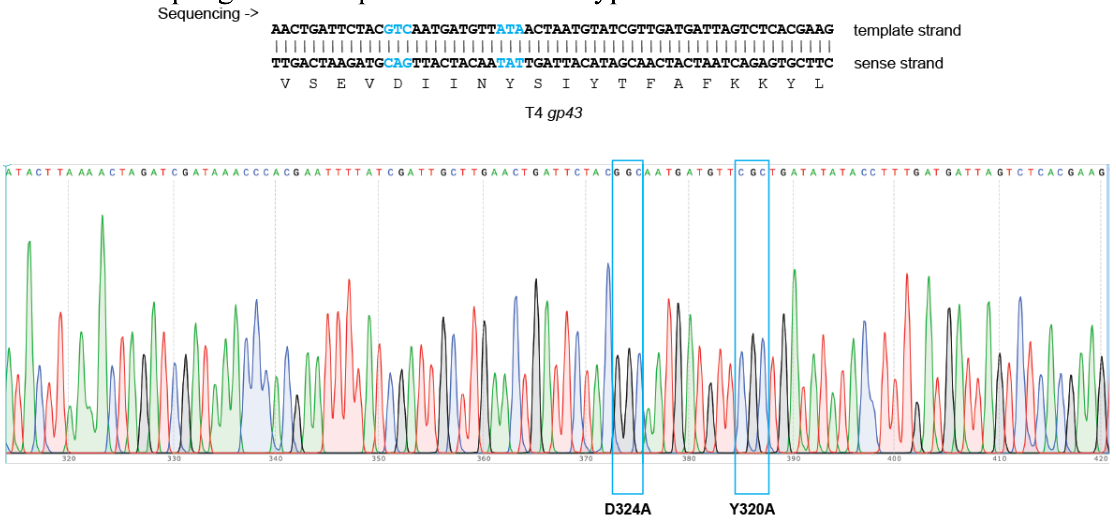

Supplementary Figure 6. Sequence of the T4 DNA polymerase exonuclease gene region comprising the mutations introduced in T4DNApolMut01. Top panel shows the sequence of the wild-type T4 phage with the direction of sequencing indicated. Mutated nucleotides are in blue. The bottom panel shows the sequence chromatogram corresponding to the mutant phage T4DNApolMut01.

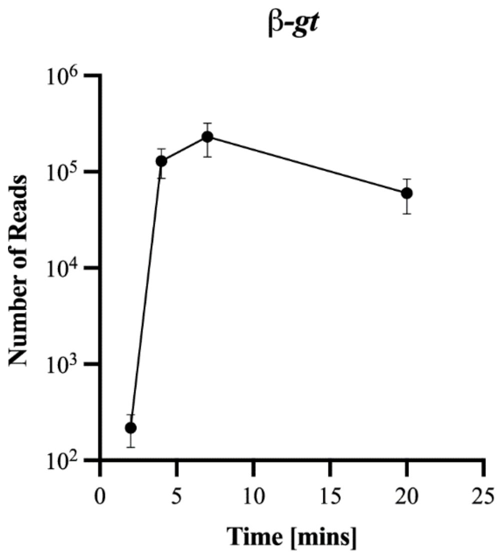

Supplementary Figure 7. *β-gt* expression level during T4 infection cycle. Number of *β-gt* reads plotted against time. Data derived from raw sequencing data of Wolfram-Schauerte et al ., (1). Data shown are the mean of three biological replicates, represented as mean value ± SD.

```
TATGTAGGAAAATACGCTTAATCGTTTAACATAAA--AGGAAT--AATATG   β-gt (wildtype T4)
||||||||||||||||||||||||||||||||  |  ||||||  ||||||
TATGTAGGAAAATACGCTTAATCGTTTAACAT--AGGAGGAATTCAATATG   mVenus NB
```

Supplementary Figure 8. Upstream sequence of *mVenusNB* in comparison with that of *β-gt*. Nucleotide comparison of the upstream sequence of *β-gt* in wild-type T4 and the intended sequence, with modification to the SD sequence (highlighted in red), to be included during insertion of *mVenusNB* gene. The start codon of β-gt and mVenus NB are shown in blue.

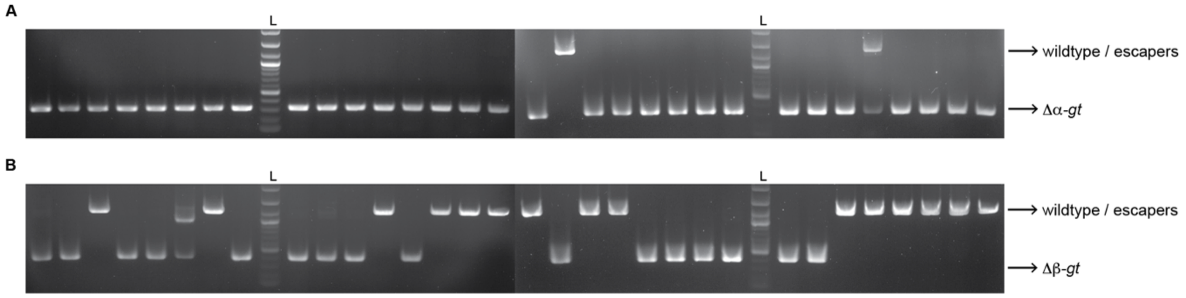

Supplementary Figure 9. PCR analysis of single plaques of lysis obtained by the fast genome editing protocol. A. PCR products of T4 single plaques obtained upon direct plating on the selection strain (DH10B/pCas13b-Spc(*α-gt*)), after one hour of incubation in the recombination strain (DH10B/pBAD24-HA(*α-gt*)). Arrow pointing to Δ αgt indicates the fragment size expected with the intended deletion. Arrow pointing to wild-type/escapers indicates the size expected with the wild-type gene. L: 1kb+ DNA ladder. **B.** Similar to A, but with the selection and recombination strain corresponding to *β-gt* deletion.

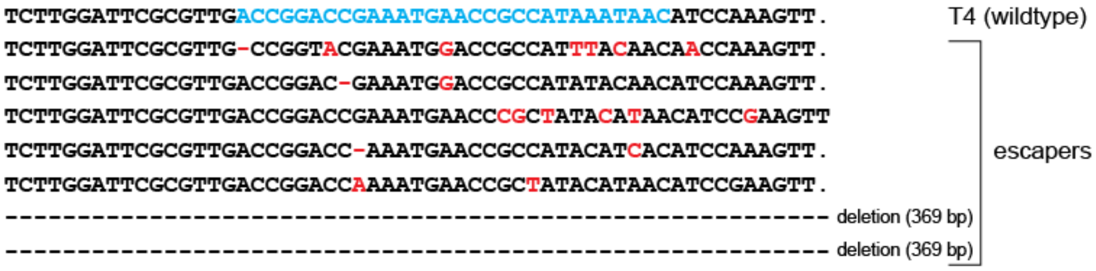

Supplementary Figure 10. CRISPR-Cas13b targeting gives rise to T4 escape mutants. Sequences of escape phages obtained upon targeting of T4 with a Cas13b spacer specific to *β-gt*. Two escape mutants showed deletion across the protospacer region due to microhomology mediated recombination across the sequence TTGCCAWAA, while other contain several point mutations.

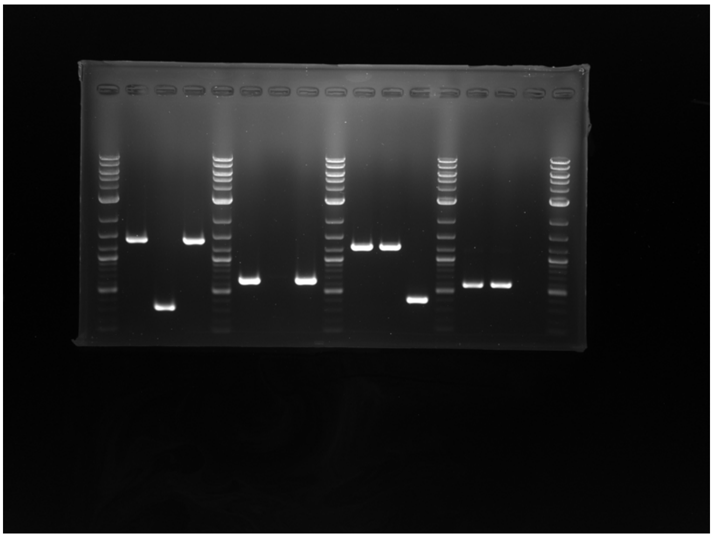

Supplementary Figure 11. Original uncropped image - PCR confirmation of the purified recombinant phages T4Δ*α-gt* and T4Δ*β-gt*.

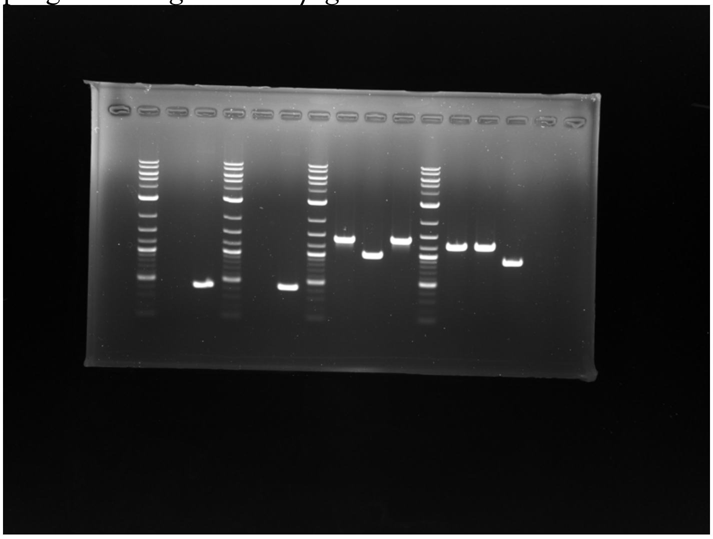

Supplementary Figure 12. Original uncropped image - PCR confirmation of the presence of the desired mutations in T4DNApolMut01 (left half of the gel) and replacement of *β-gt* with *mVenusNB* (right most four lanes, including ladder).

Supplementary Table 1: Cas13 spacers used in this study

Type VI – subtype	Target gene	Sequence (5′ → 3′)
VI-A	*α-gt*	TTACACCACAACCTTCAAGACCTCGAGCCA
VI-A	*β-gt*	CCATAAATTTTTGCGCAGATAAAATTGCTA
VI-B	*α-gt*	ATTCCATATCATTTTCATCAAACCAAATGA
VI-B	*β-gt*	AATAGAAAGCATCTCTTTACGCAAAACATC
VI-B	*gp43* (*DNApol*)	TAACTAATGTATCGTTGATGATTAGTCTCA
VI-B	*denA*	TATACTTCCATTGTCGTCTAATTCTAGCTC
VI-B	*Alc*	TCCCGTCAAAGGATTAACGATGATATAGCA
VI-B	*gp56*	GTTCTTCAGATAATAAAGTTTAAAGATTTC
VI-B	*gp43* (*DNApol*)	AACTCTGCTGAGTTTCCATACCCATGATTT
VI-B	*gp43* (*DNApol*)	AGGAATAACCTTATGTTCACCTTTCAATGA
VI-B	*res*	TCGTAATGAATGCACAACTTTTTCTAGTTC
VI-B	*res*	CCTCTACATATAGCTGATTTGCATATTGAA
VI-B	*dexA*	GAAGAGATAGAGGATCGCATTCTTCCTCTG
VI-B	*gp3*	CAGTACATACTAAAGCCGGGTCTGAATCTT
VI-B	*gp7*	GAGATAATGGATCAGAATCTATAGGTGCAT
VI-B	*gp61*	CCTCAGGATAAGCTTTGATGGTGATATATT
VI-B	*gp6*	AACCAGCATGAACCATTGACTTTCTCGTCC
VI-B	*gp6*	GGTTTCGCGTTTAATAGTACCCAATTCGCG
VI-B	*Vs. 6*	ACCACCTTCAATTTTAACTGTAGGTTGTGG
VI-B	*gp15*	GTCTATTTACATTACTAGTGAAAGTTTGTT
VI-B	*gp10*	TAATCATTAACTGTACCCTTTGGAAGATTT
VI-B	*gp10*	GCGGAGTGTGAGTAGAGTTAGTAGATGCTT
VI-B	*gp34*	GGTTAAGGTTAGTGAACCATTAACCGTCTG
VI-B	*hoc*	TCGCTTCAGCCGTTTCCGGGCCTCCTTCAG
VI-B	*rIIA*	TATTAATACCAAACAGACGATAACTTGATG
VI-B	*gp18*	CACAAACTCATTTCTATCAATTACTGACG
VI-B	*gp18*	TATCGCAAACTACACGATATTCATAAATTC
VI-B	*gp12*	ACGATGCATCAGGAACTCCATTTACTCCAG
VI-B	*gp12*	CCAACGTTGCTGGTGTAACTGCTTTGGTAT
VI-B	*uvsW*	GAACAATGATAAGAATTTTACCTTCATAAT
VI-B	*gp42*	CCAAATCTTCGGTGTTTCACCCGGAATATC
VI-B	*dda*	ATGTAGGAAGTGCTTTCACTTTACTAAACT
VI-B	*gp30.3*	TTCAACATTTTTCACCTTACACCCTTGGAG
VI-B	*gp23*	GTAGCATTGTAACCGTGGTCACCACCGATT

Supplementary Table 2: Primers used in this study

Plasmid construct	Oligonucleotide	Sequence (5′ → 3′)
pC0003-Spc(*α-gt*)	T4_*α-gt*_Cas13a_for	AAAC *TTACACCACAACCTTCAAGACCTCGAGCCA*
	T4_*α-gt*_Cas13a_rev	TATC *TGGCTCGAGGTCTTGAAGGTTGTGGTGTAA*
pC0003-Spc(*β-gt*)	T4_*β-gt*_Cas13a_for	AAAC *CCATAAATTTTTGCGCAGATAAAATTGCTA*
	T4_*β-gt*_Cas13a_rev	TATC *TAGCAATTTTATCTGCGCAAAAATTTATGG*
pCas13b-Spc(*α-gt*)	T4_*α-gt*_Cas13b_for	ACAAC *ATTCCATATCATTTTCATCAAACCAAATGA*
	T4_*α-gt*_Cas13b_rev	CAAC *TCATTTGGTTTGATGAAAATGATATGGAAT* G
pCas13b-Spc(*β-gt*)	T4_*β-gt*_Cas13b_for	ACAAC *ACCGGACCGAAATGAACCGCCATAAATAAC*
	T4_*β-gt*_Cas13b_rev	CAAC *GTTATTTATGGCGGTTCATTTCGGTCCGGT* G
pBAD24-HA(*α-gt*)	T4_*α-gt*_HA_for	ACAGCCAAGCTTGCATGCCTGTTCTTTAAAGCAGAAGCTTGAATCTTG ATGCTGATACAAAAATTCATATGCTTTTTCTCGCTCACGGTCATAAAGA GCTCGGTCAGCTCGAGCCAT
	T4_*α-gt*_HA_rev	GAGGAATTCACCATGGTACCCGTTTTATAGAAAATAAAATATTATTTAC ATGATTTATTAAATGAAAAGAGGAAAACTATGCGTATTTGCATTTTTAT GGCTCGAGCTGACCGAGCT
pBAD24-HA(*β-gt*)	T4_*β-gt*_HA_for	ACAGCCAAGCTTGCATGCCTGTTTTGTGAAATTTTTTAAATGGAAAGA TACCATCCGTTGTAGTTGCTTTTTCTTACAACTTTACGAAGGTCTTCTC TGTCACCGACACTGTTCGAT
	T4_*β-gt*_HA_rev	GAGGAATTCACCATGGTACCCGACATAAAGGAAAGTTTAAATGCAGA AAACGAATCCTGGGTTACAGAGACTATTTCAGATTCCGACATTTACCC TATCGAACAGTGTCGGTGACAGAG
T4 Δ*α-gt* check	T4_*α-gt*_chk_for	ATCCAACATGCTCTAGTGAATAG
	T4_*α-gt*_chk_rev	GATATGTTTGGGCCTATTCGTA
	T4_*α-gt*_chk_in_for	TATCAATCTCACGATTACCGTA
	T4_*α-gt*_chk_in_rev	TACAAGTTCTCATGACCACAA
T4 Δ*β-gt* check	T4_*β-gt*_chk_for	TCCTAACATTTATTCCACCGGT
	T4_*β-gt*_chk_rev	CCTACATGTGTATTCTCGTCAT
	T4_*β-gt*_chk_in_for	CTCATTGACTCTATCAATGAGTTC
	T4_*β-gt*_chk_in_rev	TTGTACACTGAAGAAGAGCTAT
pCas13b-Spc(*pol*Y320)	T4_*DNApol* Y320_for	ACAAC *TAACTAATGTATCGTTGATGATTAGTCTCA*
	T4_*DNApol* Y320_rev	CAAC *TGAGACTAATCATCAACGATACATTAGTTA* G
pBAD24-HA(*pol*Y320)	T4_*DNApol*_Y320A/ D324A_HA_for	ACAGCCAAGCTTGCATGCCTGCTTAAAACTAGATCGATAAACCCACG AATTTTATCGATTGCTTGAACTGATTCTACGGCAATGATGTTCGCTGAT ATATACCTTTGATGATTAGTCTCA
	T4_*DNApol*_Y320A_HA_re v	GAGGAATTCACCATGGTACCCGTCAGTTGCTCAACATGAAACCAAAA AAGGTAAATTACCATACGACGGTCCTATTAATAAACTTCGTGAGACTA ATCATCAACGATATATATCAGC
DNApol Y320AD324A mut check	T4_*DNApol*_chk_for (for sequencing)	ATTCGGAGAACAAGAATATTCATCAC
	T4_*DNApol*_chk_rev (for sequencing)	GATGAAGCGAATGGAAGACATCG
	*DNApol*Y320Amut_chk_rev	CTAATCATCAACGATATATATCAGCGA
pBAD24-HA(*β-gt*-*mVenusNB*)	*mVenusNB*-*β-gt*-For	TATGTAGGAAAATACGCTTAATCGTTTAACATAGGAGGAATTCAATATG AGTAAAGGAGAAGAACTTTTCAC
	mVenusNB-*β-gt* -Rev	AATAATAGTTCATAATTTTTATTTGTATAGTTCATCCATGCCAT

Sequences corresponding to Cas13 spacers are italicized.

Supplementary Table 3: Bacterial strains used in this study

Strain name	genotype	note
DH10B	F^-, mcrA, Δ(*mrr*-*hsd*RMS-*mcr*BC),Φ80d*lac*ZΔM15Δ*lac*X74, *end*A1, *rec*A1, *deo*R, Δ(*ara*,*leu*)7697, *ara*D139, *gal*U, *gal*K, *nup*G, *rps*L, $λ^-$	(2)
MS102	*MG1655 [rph-1, λ-,* pRNA1::tdCherry)	This study

Supplementary Table 4: Phage strains used in this study

Strain name	genotype	note
T4Δ*α-gt*	Δ*α-gt*	This study
T4Δ*β-gt*	Δ*β-gt*	This study
T4DNApolMut01	*gp43Y320AD324A*	This study
T4Δ*β-gt*∇*mVenus*	Δ*β-gt* ∇*mVenus*	This study

REFERENCES

1. Wolfram-Schauerte M, Pozhydaieva N, Viering M, Glatter T, Hofer K. 2022. Integrated Omics Reveal Time-Resolved Insights into T4 Phage Infection of E. coli on Proteome and Transcriptome Levels. Viruses 14.
2. Durfee T, Nelson R, Baldwin S, Plunkett G, 3rd, Burland V, Mau B, Petrosino JF, Qin X, Muzny DM, Ayele M, Gibbs RA, Csorgo B, Posfai G, Weinstock GM, Blattner FR. 2008. The complete genome sequence of Escherichia coli DH10B: insights into the biology of a laboratory workhorse. J Bacteriol 190:2597-606.
